# Analysis of senescence in gingival tissues and gingival fibroblast cultures

**DOI:** 10.1002/cre2.581

**Published:** 2022-05-02

**Authors:** Masae Furukawa, Kazunari Matsuda, Yu Aoki, Mitsuyoshi Yamada, Jingshu Wang, Maki Watanabe, Mie Kurosawa, Yosuke Shikama, Kenji Matsushita

**Affiliations:** ^1^ Department of Oral Disease Research National Center for Geriatrics and Gerontology Obu Japan; ^2^ Daiichi Sankyo Healthcare Co., Ltd. Tokyo Japan; ^3^ Department of Operative Dentistry, School of Dentistry Aichi Gakuin University Nagoya Japan

**Keywords:** aging, cytokines, hydrogen peroxide, inflammation

## Abstract

**Objective:**

To determine senescence‐associated changes in the gingival tissues of aged mice and gingival fibroblast cultures.

**Materials and Methods:**

The production of senescence‐associated β‐galactosidase (SA‐β‐gal) and mRNA expression of p16, p21, interleukin (IL)‐1β, and tumor necrosis factor α (TNF‐α) were evaluated in gingival tissues, gingival fibroblasts of 10‐ and 20‐month‐old C57BL/6NCrl mice, and multiple‐passaged and hydrogen peroxide‐stimulated human gingival fibroblasts (HGFs). Changes in molecular expression in HGF cultures due to senescent cell elimination by the senolytic drug ABT‐263 (Navitoclax) were analyzed.

**Results:**

Compared to 10‐week‐old mice, the 20‐month‐old mice had higher numbers of M1 macrophages. The proportion of cells expressing SA‐β‐gal were also higher in 20‐ month‐old mice than in 10‐week‐old‐mice. Gingival fibroblasts in 20‐month‐old mice expressed less collagen 1a1, collagen 4a1, and collagen 4a2 mRNA than those in 10‐week‐old mice. Compared to control cells, H2O2 treated HGF cells expressed higher levels of SA‐β‐gal and p16, p21, IL‐1β, and TNF‐α. Furthermore, ABT‐263 suppressed HGF cell expression of cytokines after senescence induction.

**Conclusions:**

Senescence‐associated changes were observed in the gingival tissues of aged mice and HGF cultures. In addition, the potential of senolytic drugs to modify aging‐related changes in the gingiva was shown.

## 1 INTRODUCTION

Aging is the most powerful risk factor for chronic diseases and increased mortality (Global 2015 DALYs & HALE Collaborators, 2016). It has been suggested that aging also contributes to the development of lifestyle‐related diseases, such as cardiovascular diseases, diabetes, and obesity. In addition, the pathogenesis of lifestyle‐related diseases may be attributed to the aging of cells in blood vessels and adipose tissues (Shimizu et al., 2014; Ungvari et al., 2018). Aging is also considered a risk factor for oral mucosal diseases, such as chronic periodontitis, and the incidence of periodontitis has been shown to increase with age (Darveau, 2010).

Cellular senescence (Coppe et al., 2010) not only affects an individual's aging process but may also be involved in the development of age‐related diseases. It is a phenomenon (Hayflick, 1965) of irreversible cell cycle arrest caused by telomere shortening, oncogene and tumor suppressor gene regulation, and radiation and reactive oxygen species‐induced stresses. Senescent cells share common characteristics, including cell cycle constancy, cell enlargement and flattening, formation of senescence‐associated heterochromatic foci, formation of DNA damage foci (DNA‐SCARS), activation of senescence‐associated β‐galactosidase (SA‐β‐gal), and increased expression of cell cycle inhibitors p16 and p21 (Kuilman et al., 2010). Senescent cells exhibit a phenomenon in which various inflammatory cytokines and physiologically active substances, such as matrix metalloproteases and growth factors (senescence‐associated secretory phenotype [SASP]) are secreted, and they function to maintain homeostasis within the tissue microenvironment, such as in wound healing (Acosta et al., 2008; Kuilman et al., 2008; Wajapeyee et al., 2008). In contrast, this phenomenon causes chronic inflammation in the surrounding tissues, with suggested involvement in the onset and progression of age‐related diseases, such as cancer, arteriosclerosis, chronic obstructive pulmonary disease, and Alzheimer's disease (Watanabe et al., 2017). There have been reports on the use of hydrogen peroxide (H_2_O_2_) to induce gingival fibroblast senescence (Kiyoshima et al., 2012; Xia et al., 2017) and the effects of senescence of alveolar bone cells on periodontal disease (Aquino‐Martinez, Khosla, et al., 2020; Aquino‐Martinez, Rowsey, et al., 2020). Some researchers have also evaluated periodontal ligament cells from donors of different ages (Wu et al., 2015). On the other hand, conventional age‐related changes in periodontal tissues have been analyzed morphologically (Van der Velden, 1984). In the elderly, the oral mucosa becomes atrophic. The epithelial tissue becomes thinner, the connective tissue loses its elasticity, and the number of capillaries decreases, resulting in a reduced blood supply. Clinically, the gingiva recedes and becomes less elastic. In addition, a decrease in keratinization and in the number of fibroblasts has been observed in gingival tissue with age (Andreescu et al., 2013). On the other hand, age‐related changes in gingival tissue have not been fully analyzed from the molecular and cellular biological perspectives.

Therefore, in this study, we examined the molecular dynamics of senescence‐related changes in gingival tissues using an aged mouse model and the expression of senescence‐ and inflammation‐related genes in gingival fibroblast cultures. In addition, the possibility of controlling gingival senescence using a senolytic drug was investigated in gingival fibroblast cultures.

## 2 METHODS

### 2.1 Cell culture

Normal human gingival fibroblasts (HGFs; #PCS‐201‐018; American Type Culture Collection, Manassas, VA, USA) were cultured in Dulbecco's Modified Eagle Medium (DMEM; #D6429; Sigma‐Aldrich, St. Louis, MO, USA) with 10% fetal bovine serum (FBS) at 37°C in a humidified atmosphere of 5% CO_2_ (Hsu et al., 2019).

The HGFs had been obtained from a 60‐year‐old Caucasian woman. The population doubling levels of the cells were 15. The cells were received after two passages (passaged three times before the experiment) and cultured until 70% confluence in 100‐mm diameter culture dishes. They were then transferred to six‐well flat‐bottom culture plates for subsequent experiments. Senescence of HGFs was induced by multiple passages or by stimulation with H_2_O_2_ (Kiyoshima et al., 2012).

### 2.2 Senescence of HGFs induced by multiple passages

HGFs at a density of 2 × 10^5^ cells per dish were seeded in 100‐mm tissue culture dishes. HGFs were passaged when the culture reached approximately 60%–75% confluency. For SA‐β‐gal staining, 1500 fibroblasts were placed in a 35 × 10 mm dish for each passage; 10 μM of the senolytic drug ABT‐263 (Navitoclax, ChemScene, CS‐0013) was added to nine‐generation passage‐cultured HGFs, and the cells were incubated for 24 h. At the end of the incubation period, total RNA was collected, and the expression of p16, p21, interleukin (IL)‐1β, and tumor necrosis factor α (TNF‐α) was examined by real‐time polymerase chain reaction (PCR).

### 2.3 Senescence of HGFs induced by H_2_O_2_

At the fifth passage, HGFs were placed in a 35 × 10 mm dish. On Day 0, the cells were stimulated with 20 μM H_2_O_2_ (Fujifilm Wako, Osaka, Japan) (Kiyoshima et al., 2012). Five days after stimulation with H_2_O_2_, the cells were harvested for SA‐β‐gal staining and assessed for SA‐β‐gal activity, and real‐time PCR was performed.

### 2.4 Animals

Male C57/B6N mice aged 10 weeks and 20 months (*n* = 18 per group) were used. The animals were bred in a strictly monitored air‐conditioned clean room and were given standard laboratory pellets and water ad libitum at the National Center for Geriatrics and Gerontology. The animals were sacrificed, and gingival tissues were collected for real‐time PCR and primary gingival fibroblast culture. All experimental procedures were reviewed and approved by the National Center for Geriatrics and Gerontology.

The protocol of this animal study was approved by the Research Facilities Committee for Laboratory Animal Science at the National Center for Geriatrics and Gerontology (approval number 30–58) and the study was carried out in accordance with the current version of the Act on Welfare and Management of Animals (1973).

### 2.5 Immunofluorescence analysis

Following fixation with 4% paraformaldehyde, the oral mucosa around the upper maxillary molars was permeabilized and blocked with Blocking One (03953‐95; Nacalai Tesque, Kyoto, Japan). The sections were incubated with Anti‐Iba1 antibody (1:1000 dilution, ab5076; Abcam, Cambridge, UK) and anti‐inducible Nitric Oxide Synthase (iNOS) antibody (1:500 diluent, ab178945; Abcam) or with Anti‐Iba1 antibody (1:1000 dilution, ab5076; Abcam) and anti‐mannose (CD206) receptor antibody (1:500 diluent, ab64693; Abcam) overnight at 4°C. Following incubation, the sections were washed with phosphate‐buffer solution (PBS).

The secondary antibodies were Alexa Fluor 594 chicken anti‐rabbit IgG(H + L) (1:500; Invitrogen A21442) for anti‐iNOS antibody and anti‐mannose receptor antibody and Alexa Fluor 488 donkey anti‐goat IgG (H + L) (10 μg/ml; Invitrogen A11055, USA) for Anti‐Ibal1 antibody, and the sections were counterstained with 4′, 6‐diamidino‐2‐phenylindole (SJ217, Dojindo; Kumamoto, JP). The samples were observed under a fluorescence microscope (BZ‐9000; Keyence, Osaka, Japan). The images shown are representative of at least three separate experiments (Figure 2b).

### 2.6 Primary mouse gingival fibroblast culture

The oral mucosa around the upper maxillary molars was immediately washed with PBS and transferred to a culture dish. Mouse gingival fibroblasts were grown in DMEM supplemented with 10% FBS. The medium was changed every other day. The cells were grown to semiconfluence, harvested by trypsinization at 37°C for 3 min, and then subcultivated with DMEM supplemented with 10% FBS in a new dish. Cells that had been passaged twice were used as early passage fibroblasts (Iwayama et al., 2012).

### 2.7 Measurement of SA‐β‐gal

Cells in which senescence was induced by multiple passages and stimulation by H_2_O_2_ were assessed by SA‐β‐gal staining using a senescence detection kit (Bio Vision, CA, USA) following the manufacturer's instructions. Cellular SA‐β‐gal activity was also evaluated using a 96‐well cellular senescence assay kit (Cell Biolabs, San Diego, CA, USA) following the assay protocol (Yi et al., 2017). Positively stained cells were counted under a x20 magnification microscope in three random fields for each experimental condition (Dimri et al., 1995).

### 2.8 Real‐time PCR

Total RNA was isolated from gingival tissues and HGFs using Nucleospin RNA (Takara Bio Inc., Shiga, Japan; cat no. U0955C) according to the manufacturer's instructions. The total RNA concentration was corrected to 100 ng/μl using the NanoDrop™ 2000 spectrophotometer (Thermo Fisher, Tokyo, Japan). First‐strand cDNA synthesis was conducted using a ReverTra Ace‐α kit (Toyobo, Osaka, Japan). Real‐time PCR was performed using FastStart Essential DNA Green Master (Roche, Mannheim, Germany) according to the manufacturer's protocol. One microgram of RNA was reverse‐transcribed using the LightCycler® 96 System (Roche, Germany); the primer sequences are listed in Table 1.

**Table 1:** Sequences of primers used for real‐time polymerase chain reaction

Name	Forward	Reverse
Human p16	CTCGTGCTGATGCTACTGAGGA	GGTCGGCGCAGTTGGGCTCC
Human p21	CCGAAGTCAGTTCCTTGTGG	CATGGGTTCTGACGGACAT
Human IL‐1β	GCAGCCATGGCAGAAGTACCTGA	CCAGAGGGCAGAGGTCCAGGTC
Human TNF‐α	AGGCGCTCCCCAAGAAGACA	TCCTTGGCAAAACTGCACCT
Human MMP3	CAAAACATATTTCTTTGTAGAGGACAA	TTCAGCTATTTGCTTGGGAA
Human GAPDH	TGTCAGTGGTGGACCTGACCT	AGGGGAGATTCAGTGTGGTG
Mouse p16	CGTACCCCGATTCAGGTGAT	TTGAGCAGAAGAGCTGCTACGT
Mouse p21	GTGGGTCTGACTCCAGCCC	CCTTCTCGTGAGACGCTTAC
Mouse iNOS	CGAAACGCTTCACTTCCAA	TGAGCCTATATTGCTGTGGCT
Mouse CD206	CTCTGTTCAGCTATTGGACGC	CGGAATTTCTGGGATTCAGCTTC
Mouse Iba1	CTTTTGGACTGCTGAAGGC	GTTTCTCCAGCATTCGCTTC
Mouse Col1a	CAATGGCACGGCTGTGTGCG	AGCACTCGCCCTCCCGTCTT
Mouse Col4a	CAGATTCCGCAGTGCCCTA	GGAATAGCCGATCCACAGTGAG
Mouse Col4a2	GACCGAGTGCGGTTCAAAG	CGCAGGGCACATCCAACTT
Mouse GAPDH	AACCTGCCAAGTATGATGA	GGAGTTGCTGTTGAAGTC

Samples were normalized to the housekeeping gene *GAPDH*, and the results are shown for each sample relative to the control. The experiments were conducted in triplicate for each condition. Each value is presented as the fold change between the samples, which was determined using the ΔΔ*C*_t_ method (Yamada et al., 2018).

### 2.9 Statistical analyses

All values are presented as mean ± standard error of the mean (SEM). One‐way analysis of variance (ANOVA) and two‐way ANOVA were used to evaluate the differences between groups. When significant effects were detected, subsequent post hoc analysis was performed using Tukey's post hoc test, where *p* < .05 was considered significant. All statistical analyses were performed using EZR (Kanda, 2013) for the R software. This is a modified version of the R commander designed to add statistical functions and is frequently used in biostatistics.

## 3 RESULTS

### 3.1 Senescence‐associated changes in gingival tissues of aged mice

**Figure 1:**
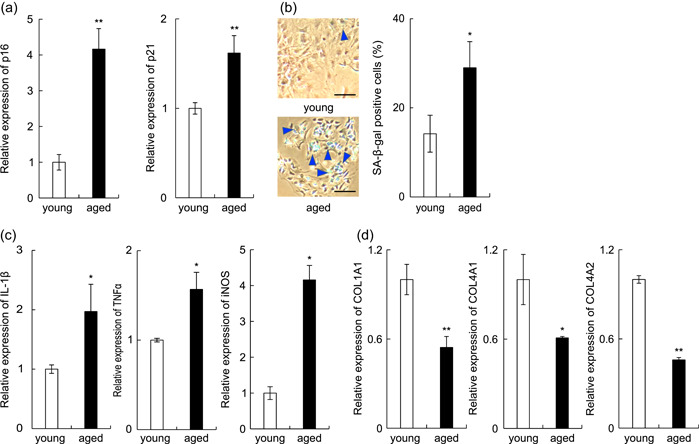
Comparison between gingival tissues of aged and young mice. (a) Total RNA was isolated from the gingival tissues of 6‐week‐old and 20‐month‐old male mice. The mRNA expression of p16 and p21 in the samples was measured by real‐time polymerase chain reaction (PCR). (b) Fibroblasts isolated from the gingival tissues of the mice were cultured in vitro. The degree of senescence of the cells passaged twice was evaluated by SA‐β‐gal staining. (c) The mRNA expression of interleukin (IL)‐1β, tumor necrosis factor α (TNF‐α), and iNOS in the samples was measured by real‐time PCR. (d) The mRNA expression of collagen 1a1, 4a1, and 4a2 in the samples was measured by real‐time PCR. *n* = 5 ± SEM, *p < .05; **p < .01 versus young mice. Image C is representative.

To investigate the senescence‐associated changes in gingival tissues, we collected oral mucosa around the upper maxillary molars from aged mice (20‐month‐old mice) and examined the expression of aging‐related molecules (SA‐β‐gal, p16, and p21) in the tissues and compared them with those in tissues from young mice. First, we examined the mRNA expression of p16 and p21 using real‐time PCR. The mRNA expression of p16 and p21 was higher in the gingival tissues of aged mice than in the gingival tissues of young mice (Figure 1a).

SA‐β‐gal expression was also greater in the gingival tissues of aged mice than in those of young mice (Figure 1b). It is known that the expression and secretion of inflammatory mediators are increased in senescent cells (Rodier & Campisi, 2011). Therefore, we compared the expression levels of IL‐1β, TNF‐α, and iNOS in gingival tissues of aged mice with those in gingival tissues of young mice. The mRNA expression of IL‐1β, TNF‐α, and iNOS was significantly greater in the gingival tissues of aged mice than in young mice (Figure 1c). To further investigate the relationship between aging and wound healing, we examined the expression of collagen in the same tissues. The mRNA expressions of collagen 1a1, collagen 4a1, and collagen 4a2 in gingival tissues from aged mice were markedly reduced relative to those in gingival tissues from young mice (Figure 1d).

### 3.2 Senescence‐associated changes in M1/M2 macrophages in the gingiva of mice

**Figure 2:**
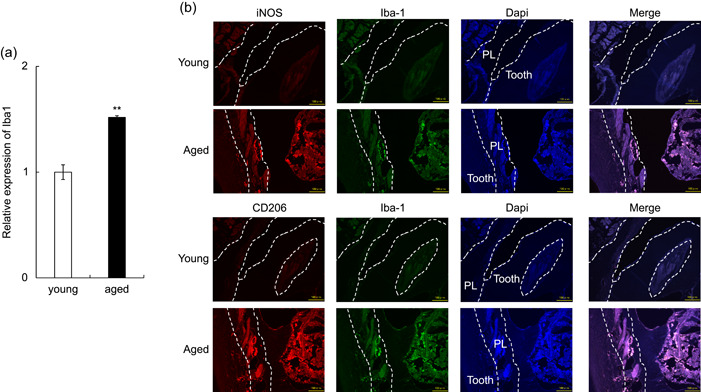
Senescence‐associated changes in M1/M2 macrophages in mice gingiva. (a) Total RNA was isolated from gingival tissues of 6‐week‐old and 20‐month‐old male mice. The mRNA expression of Iba1 in the samples was measured by real‐time polymerase chain reaction (PCR). (b) Paraffin sections of the periodontal tissues of 6‐week‐old and 20‐month‐old male mice were double‐stained with anti‐iNOS mAb and anti‐Iba1 mAb or CD206 mAb and anti‐Iba1 mAb, respectively (M1 macrophages: iNOS^+^ Iba1^+^; M2 macrophages: CD206^+^ Iba1^+^). PL, periodontal ligament. Scale bars = 100 μm.

It is known that the ratio of M1 and M2 macrophages (M1/M2 ratio) is altered in the gingival tissue of chronic periodontitis (Yang et al., 2018). Therefore, we examined the dynamics of macrophages in the collected mouse periodontal tissues. The mRNA expression of Iba1 was greater in aged mice (Figure 2a). In addition, CD206^+^ and Iba‐1^+^ M2 macrophages around the periodontal ligament vessels in aged mice were found to be increased compared to those around the periodontal ligament vessels in young mice (Figure 2b). The results of fluorescence immunostaining showed that cells double‐stained with iNOS and Iba‐1, a marker of M1 macrophages, in the perivascular area of the periodontal ligament were more abundant in aged mice than in young mice.

### 3.3 Senescence of gingival fibroblasts in vitro

**Figure 3:**
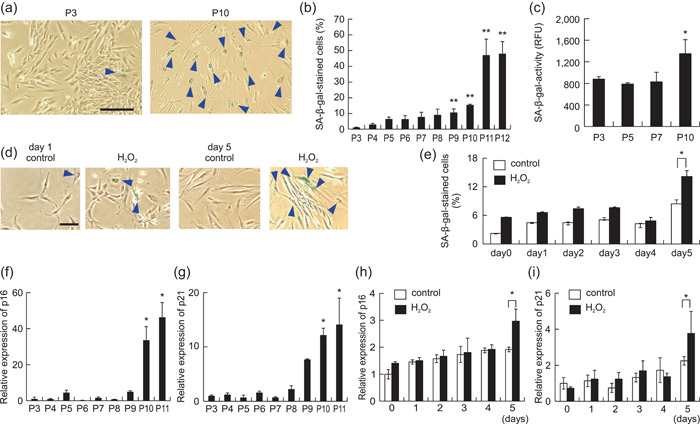
Analysis of cellular senescence of human gingival fibroblasts (HGFs) in culture. (a,b) Multiple passages of HGFs were executed in vitro. Senescence‐associated β‐galactosidase (SA‐β‐gal) staining was performed for HGFs of each passage to evaluate the degree of senescence. The images show a typical example (P3 vs. P10). Scale bars = 50 μm. (c) SA‐β‐gal activity in HGFs of multiple passages was measured. The images show a typical example (control vs. H_2_O_2_). Scale bars = 50 μm. (d,e) HGFs were treated with 20 µM H_2_O_2_ for 5 days. SA‐β‐gal staining of HGFs was performed to evaluate the degree of senescence. The mRNA expression of p16 and p21 in multiple‐passaged HGFs (f,g) and H_2_O_2_‐treated HGFs (h,i) was measured by real‐time polymerase chain reaction. *n* = 5 ± SEM, *p < .05; **p < .01 versus P3 (b,c,f,g). *n* = 5 ± SEM, *p < .05; **p < .01 versus control (e,h,i). (a,d) are representative images.

We investigated the senescence properties of gingival fibroblasts in vitro. Cells are thought to undergo senescence by reaching the limit of the number of possible divisions. Cellular senescence is also induced by oxidative stress. Therefore, the properties of gingival tissue‐derived cells with induced senescence were examined by the long‐term subculture of HGFs or by applying oxidative stress to the cells. First, senescence of cultured HGFs was assessed by SA‐β‐gal staining and measurement of SA‐β‐gal activity. A significant increase in the proportion of SA‐β‐gal‐positive cells was observed after nine passages of HGFs (Figure 3a,b). The proportion of SA‐β‐gal‐positive cells continued to increase until passage 12, after which the cells stopped proliferating. An increase in SA‐β‐gal activity was also observed in HGFs after 10 passages (Figure 3c).

Next, HGFs were incubated for 5 days after the addition of 20 μM H_2_O_2_, and the degree of senescence was evaluated using SA‐β‐gal staining. A significant increase in the proportion of SA‐β‐gal‐positive cells was observed in the culture on Day 5 after stimulation with 20 μM H_2_O_2_ (Figure 3d,e). Subsequently, we examined the expression of p16 and p21, which are aging markers associated with the cell cycle, in long‐term subcultures and H_2_O_2_‐stimulated HGFs. A significant increase was observed in the mRNA expression of p16 and p21 in HGFs after 10 and 11 passages (Figure 3f,g). In addition, a significant increase in p16 and p21 mRNA expression was confirmed in HGFs stimulated with 20 μM H_2_O_2_ (Figure 3h,i).

**Figure 4:**
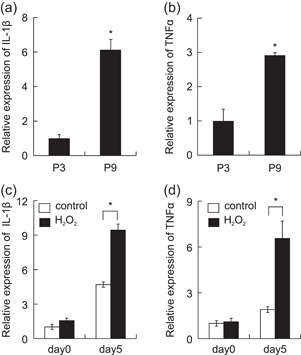
Inflammatory cytokines increase in senescent gingival fibroblast cultures. The mRNA expression of interleukin (IL)‐1β and tumor necrosis factor α (TNF‐α) in ninth‐passaged human gingival fibroblasts (HGFs) and H_2_O_2_‐treated HGFs (a,b) was measured by real‐time polymerase chain reaction. *n* = 5 ± SEM, *p < .05 versus P3 (a,b). *n* = 5 ± SEM, *p < .05 versus control (c,d).

We also investigated the altered expression of inflammatory cytokines IL‐1β and TNF‐α in HGF cultures with increased senescent cells. The mRNA expression of IL‐1β and TNF‐α was significantly higher in HGFs passaged nine times than in cells passaged three times (Figure 4a,b). The expression of IL‐1β and TNF‐α mRNA was significantly increased in HGFs stimulated with 20 µM H_2_O_2_ for 5 days (Figure 4c,d).

**Figure 5:**
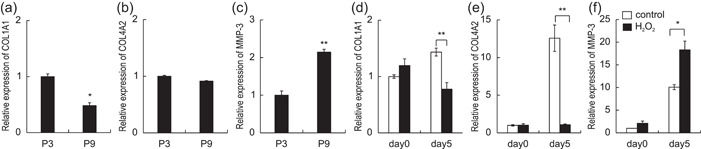
Expression of collagen‐related genes in senescent gingival fibroblast cultures. The mRNA expression of COL1A1, COL4A2, and MMP‐3 in ninth‐passaged human gingival fibroblasts (HGFs) (a–c) and H_2_O_2_‐treated HGFs (d–f) was measured by real‐time polymerase chain reaction. *n* = 5 ± SEM, *p < .05 versus P3 (a,b). *n* = 5 ± SEM, *p < .05 versus control (d–f).

Additionally, the altered expression of collagen 1a1, 4a2, and metalloproteinase‐3 (MMP‐3) was examined in HGF cultures with increased senescent cells. The mRNA expression of collagen 1a1 was significantly lower in HGFs passaged nine times than in those passaged three times (Figure 5a). The expression of col4a2 tended to decrease slightly in HGFs passaged nine times (Figure 5b). In contrast, the mRNA expression of MMP‐3 significantly increased in HGFs (Figure 5c). The same results were detected in HGFs stimulated with 20 μM H_2_O_2_ (Figure 5d–f).

**Figure 6:**
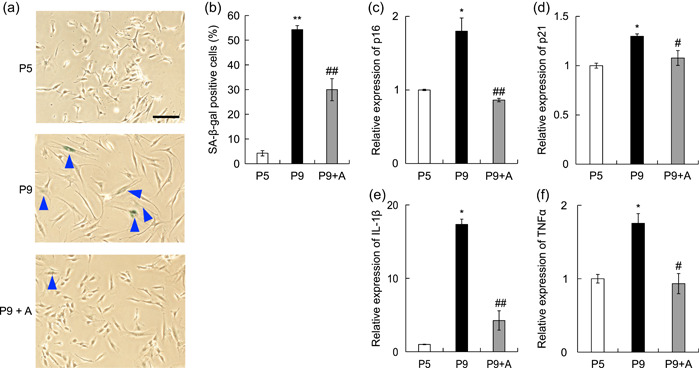
Expression of inflammatory cytokines is suppressed by ABT‐263 in aged gingival fibroblast cultures. Human gingival fibroblasts (HGFs) after nine passages were incubated with 10 µM of ABT‐263 for 24 h and then SA‐β‐gal staining of HGFs was performed to compare the degrees of senescence between P5, P9, and ABT‐263 added groups. Scale bars = 200 μm (a,b). The mRNA expression of p16, p21, interleukin (IL)‐1β, and tumor necrosis factor α (TNF‐α) was examined by real‐time polymerase chain reaction. A, ABT‐263. *n* = 5 ± SEM, *p < .05, **p < .01 versus P5 (c–f). *n* = 5 ± SEM, #*p* < .05; ##*p* < .01 versus P9 (a–e). Panel (a) shows typical examples.

To elucidate the relationship between cellular senescence and the expression of inflammatory cytokines, we analyzed changes in the expression of inflammatory cytokines when senescent cells were eliminated. First, we confirmed the effectiveness of ABT‐263 in the removal of senescent cells in HGF cultures (Aguayo‐Mazzucato et al., 2019). Ten micromolar of ABT‐263 was added to nine‐passaged HGF cultures and incubated for 24 h, following which the number of SA‐β‐gal‐positive cells was examined. There were more SA‐β‐gal‐positive cells in nine‐passaged HGF cultures than in five‐passaged HGF cultures. Treatment with ABT‐263 decreased the number of SA‐β‐gal‐positive cells in nine‐passaged HGF cultures (Figure 6a,b). Next, we examined the mRNA expression of p16, p21, IL‐1β, and TNF‐α in nine‐generation passage‐cultured HGFs treated with 10 µM ABT‐263 for 24 h. Treatment with ABT‐263 resulted in a decrease in the mRNA expression levels of p16 and p21 to their expression levels in five‐generation cultures (Figure 6c,d). Furthermore, the mRNA expression levels of IL‐1β and TNF‐α, which were increased in nine‐passaged cultures, significantly decreased with the addition of ABT‐263 (Figure 6e,f). These results suggest that increased expression of pro‐inflammatory cytokines in long‐term passaged HGFs is related to an increase in senescent cells in the culture.

## 4 DISCUSSION

The incidence of periodontitis increases with aging, and senescence is thought to be a risk factor for periodontitis (Ebersole et al., 2016). However, the relationship between senescence of periodontal tissues and the pathogenesis of periodontitis remains unclear. Conventional age‐related changes in periodontal tissues have been analyzed morphologically and histologically. However, age‐related changes in gingival tissue have not been fully examined from the molecular and cellular biological perspectives. In this study, we examined the expression of age‐related and inflammation‐related molecules using gingiva from aged mice and gingival fibroblast cultures. Expression of senescence‐associated molecules, mRNA expression of inflammatory cytokines, and the number of M1 macrophages were higher, while mRNA expression of collagen in the gingiva was lower in aged mice than in young mice. In addition, in HGF culture systems that were passaged multiple times or were stimulated with H_2_O_2_, increased mRNA expression of aging cell‐related molecules and inflammatory cytokines and decreased mRNA expression of collagen were observed, similar to the results obtained in vivo. These results suggest that in the gingiva, the expression of inflammatory molecules increases with an increase in senescent cells.

Senescence is an important risk factor for lifestyle‐related diseases, such as arteriosclerotic diseases, diabetes, and dementia (Ferencz & Gerritsen, 2015). Periodontal disease is also a lifestyle‐related disease (Genco & Borgnakke, 2013), and its relevance to systemic lifestyle‐related diseases has been reported. Periodontal tissue changes with aging (Van der Velden, 1984). The aging of periodontal tissues results in significant anatomical and histological changes that cause decreased tissue homeostasis, leading to faster disease progression and delayed tissue repair (Tsalikis, 2010; van der Velden, 1991). It is also possible that resistance to infection decreases due to a decrease in immune function in elderly people, increasing their susceptibility to periodontal disease (Ebersole et al., 2016). Further, the accumulation of senescent cells reportedly contributes to inflammation and a decrease in the function of each organ (Ovadya et al., 2018), and it has been reported that the accumulation of senescent cells contributes to an increase in inflammation, thereby reducing the function of each organ (Ovadya et al., 2018). Cellular senescence is a state in which the division and proliferation of somatic cells stop and the expression of cyclin‐dependent kinase inhibitors, such as p16 and p21, is enhanced (Baker et al., 2011, 2016). In this study, enhanced expression of p16 and p21 was confirmed in the gingival tissues of aged mice and gingival fibroblasts after multiple passages. This indicates that the cell cycle of fibroblasts constituting gingival tissues stops and the cells become senescent with aging. Increased expression of p16 and p21 also results from oxidative stress, such as that induced by H_2_O_2_ in this study (Chen et al., 2019; Macip et al., 2002; Tabasso et al., 2019). Reactive oxygen species (ROS) are strongly induced by gram‐negative bacteria, such as periodontal pathogens (Damgaard et al., 2017). Therefore, sustained induction of ROS in periodontal tissue by periodontopathic plaque may also promote gingival aging. Increased secretion of various inflammatory cytokines, chemokines, and extracellular matrix‐degrading enzymes from senescent cells has been observed, and it has been suggested that these factors cause chronic inflammation (He & Sharpless, 2017). In this study, we confirmed that aging‐related markers were expressed in gingival tissues of aged mice and that the expression of inflammatory cytokines, such as IL‐1β and TNF‐α, was also greater in their tissues.

In this study, it was confirmed that the expression of collagen 4 was attenuated together with collagen 1 in the gingiva of aged mice. Collagen 4 forms a reticular structure and is the predominant collagen in basement membranes. The expression of collagen 4 increases during basement membrane proliferation and new basement membrane formation. Since collagen 4 forms a barrier between the epithelium and the dermis, a decrease in its expression may cause pathogen invasion from the epithelium to the dermis. Decreased expression of collagen 4 in the basement membrane has been reported to cause the failure of epithelial tissue healing (Sato et al., 1999), and it may therefore be an indicator of gingival fragility in aged gingiva. Matrix metalloproteinase (MMP)‐3 (stromelysin‐1) degrades collagen in the basement membrane and induces the synthesis of other MMPs, such as MMP‐1 and MMP‐9, which can potently destroy periodontal tissue (Sorsa et al., 2004). In contrast, the association between the aging of periodontal tissue‐derived cells and the expression of MMP‐3 is unclear. In this study, the mRNA expression of MMP‐3 mRNA was also increased in aged gingival fibroblast cultures (Figure 5c,f). Increased expression of MMP‐3 in aged fibroblasts coupled with reduced collagen expression may contribute to gingival fragility.

The mRNA induction of inflammatory cytokines revealed in this study may cause SASP. It is also possible that the SASP factor acts on fibroblasts around senescent cells, leading to the secondary induction of inflammatory cytokines and increased expression. These results suggest that senescent cells in the gingival tissue may continuously express inflammatory mediators. In this study, we also clarified that there was an increase in M1 macrophages in the gingival tissues of aged mice, along with an increase in total macrophages (Figure 2). Macrophages fall into classical M1 and alternative M2 categories. M1 macrophages release various pro‐inflammatory cytokines, which are involved in enhancing the inflammatory response. In contrast, M2 macrophages are thought to be involved in the resolution of inflammation. In addition, changes in the balance of M1/M2 macrophages have been suggested to contribute to the development and progression of inflammation (Sica & Mantovani, 2012). In this study, the total number of macrophages (expression level of Iba‐1 mRNA) was significantly higher in the gingival tissues of aged mice than in the gingival tissues of young mice. However, when the M1 and M2 phenotypes were examined, it became clear that both M1 and M2 macrophages were increased in the periodontal ligaments of aged mice. In this study, the function of macrophages in the periodontal tissues of aged mice could not be investigated, and the significance of the findings is unclear. The increase in M1/M2 macrophages in aged periodontal tissues may be associated with reduced infectious immunity and enhanced inflammatory response in aged animals. We need to examine this point in the future. M1‐M2 macrophage distinction is too simplistic and barely discernible in vivo (Jablonski et al., 2015). We also speculate that this phenomenon might be due to aging as a risk factor for periodontitis. However, based on the results of this study, it is impossible to conclude the causal relationship between age‐related changes in the gingiva and the exacerbation of gingival inflammation, and the development of chronic periodontitis. In the future, we will confirm this relationship by conducting infection experiments with periodontopathic bacteria in aged mice.

The possibility of suppressing the onset of arteriosclerosis, dementia, and cancer by eliminating senescent cells has been demonstrated in mouse models (Bussian et al., 2018; Kovacovicova et al., 2018; Matjusaitis et al., 2016; Zhang et al., 2019). Reagents that induce selective cell death of senescent cells (senolytic drugs) have been reported (Kirkland et al., 2017; Zhu et al., 2015). ABT‐263 is one of them and has been shown to induce cell death, such as that of aging human fetal fibroblasts (Chang et al., 2016). Therefore, we examined the change in the expression of inflammatory cytokines following the addition of ABT‐263 to gingival fibroblasts in which senescence was induced. ABT‐263 attenuated the expression of H_2_O_2_‐induced cellular senescence markers and decreased the mRNA expression of IL‐1β and TNF‐α in the cultures. Therefore, it was thought that the increase in the expression of inflammatory cytokines by the addition of H_2_O_2_ to the cultures was due to an increase in senescent cells. Since the elimination of senescent cells is an entirely new therapeutic paradigm, the application of senolytic drugs for human intervention must be critical enough to render currently available treatments ineffective (Kirkland & Tchkonia, 2020). In the future, we would like to examine the effectiveness of senolytic drugs in treating gingival tissue.

## 5 CONCLUSION

In this study, we characterized the expression of aging and inflammation‐related molecules in the gingiva of aging mice and HGF cultures. In the future, clarifying the causal relationship between these changes and oral mucosal diseases, such as periodontitis, can contribute to the possibility of using senolytic drugs for oral mucosal diseases.

## AUTHOR CONTRIBUTIONS

All authors have made substantial contributions to the conception and design of this study. Masae Furukawa, Mitsuyoshi Yamada, Jingshu Wang, Mie Kurosawa, and Maki Watanabe were involved in data collection and analysis. Masae Furukawa, Kazunari Matsuda, Yosuke Shikama, Yu Aoki, and Kazunari Matsuda were involved in data interpretation, drafting of the manuscript, and revising it critically. All authors approved the final version of the manuscript.

## CONFLICT OF INTEREST

The authors declare no conflict of interest.

## ETHICS STATEMENT

All experimental procedures were reviewed and approved by the National Center for Geriatrics and Gerontology. The protocol of the animal study was approved by the Research Facilities Committee for Laboratory Animal Science at the National Center of Geriatrics and Gerontology (approval number 30–58) and carried out in accordance with the current version of the Act on Welfare and Management of Animals (1973).

## Data Availability

The data sets used and/or analyzed during this study are available from the corresponding authors on reasonable request.
